# Progression to end-stage renal disease due to IgG4-related nephritis: a case report

**DOI:** 10.1093/omcr/omae179

**Published:** 2025-01-18

**Authors:** Maysoun Kudsi, Raghad Tarcha, Naram Khalayli, Nour Rabah, Karam Rabah, Fatima Alzahraa Alghawe

**Affiliations:** Professor of Rheumatology, Faculty of Medicine, Damascus University, Damascus, Syria; Department of Rheumatology, Faculty of Medicine, Damascus University, Damascus, Syria; Faculty of Medicine, Damascus University, Damascus, Syria; 6^th^ year medical student, Faculty of Medicine, Syrian Private University, Syria; Faculty of Medicine, Syrian Private University, Syria; Department of Rheumatology, Faculty of Medicine, Damascus University, Damascus, Syria

**Keywords:** IgG4-related nephritis, IgG4-related kidney disease (IgG4-RKD), hemodialysis, parotid gland involvement

## Abstract

IgG4-related disease (IgG4-RD) is a rare but increasingly recognised condition that can involve multiple organs, including the kidneys which often presents as tubulointerstitial nephritis.

Treatment with glucocorticoids is the first line of therapy, but other options may be needed in refractory cases.

This case report explores a 68-year-old female, diagnosed with the patient initially responded to glucocorticoids but had a relapse, leading to progressive renal insufficiency and ultimately death.

Our case is a rare case observing the progression to end-stage kidney disease from IgG4-RD, and the first case of which the patient had died in a short period.

## Introduction

Immunoglobulin G4-related disease is an infrequent systemic condition affecting males more than females [1]. Kidney involvement happened in 25%of patients, often presenting as tubulointerstitial nephritis [2].

Fatigue, arthralgia, salivary and lacrimal gland enlargement, symptoms of pancreatitis, tubulointerstitial nephritis, and retroperitoneal fibrosis are the frequent manifestations [1].

It occurs when an auto antigen triggers an immune response characterized by increased cytokine production that induces IgG4-producing plasma cells and fibrosis, respectively [2].

Its diagnosis depends on (RCD criteria): organ involvement; serum IgG4 level of more than 135 mg/dl; and positive for 2 out of 3 pathological sub-items [3]. The absence of diagnostic criteria for IgG4 renal disease leads to a challenge in the diagnosis [2]. We reported a case of IgG4-related renal involvement, in which the patient died despite the treatment with steroids and hemodialysis.

## Case report

A 68-year-old female, non-smoker, non-alcoholic, with a past medical history of hypertension for 15 years, and hypothyroidism for 10 years, presented to the hospital with the elevation of serum creatinine, in September 2022.

**Figure 1:**
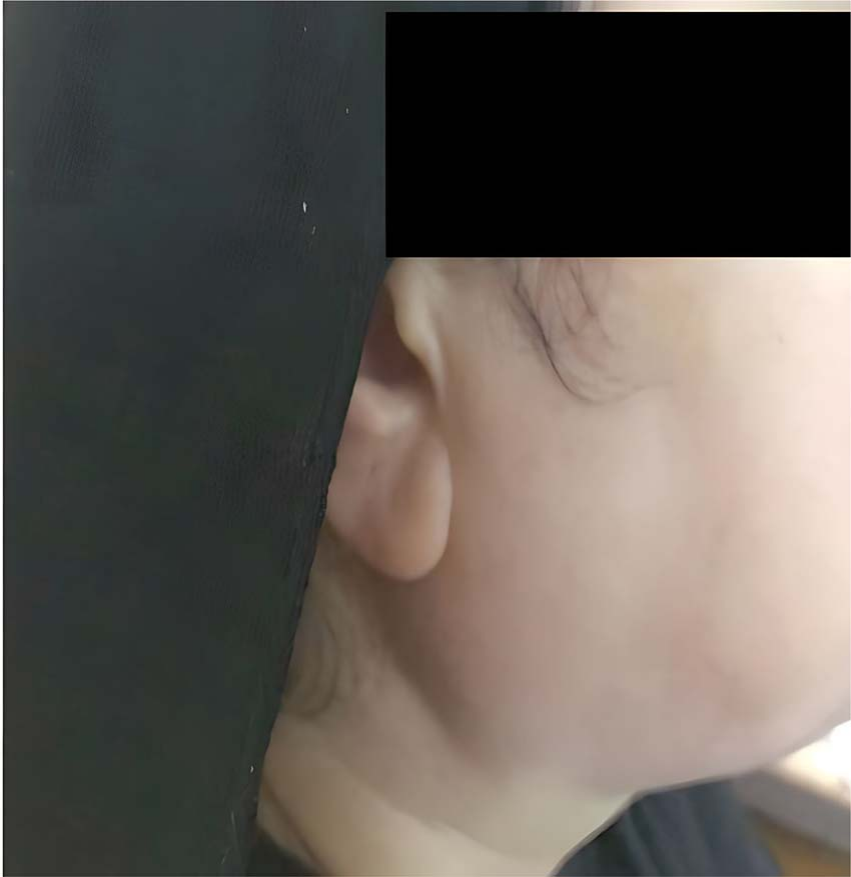
Photograph of the patient showing right parotid gland enlargement.

**Figure 2:**
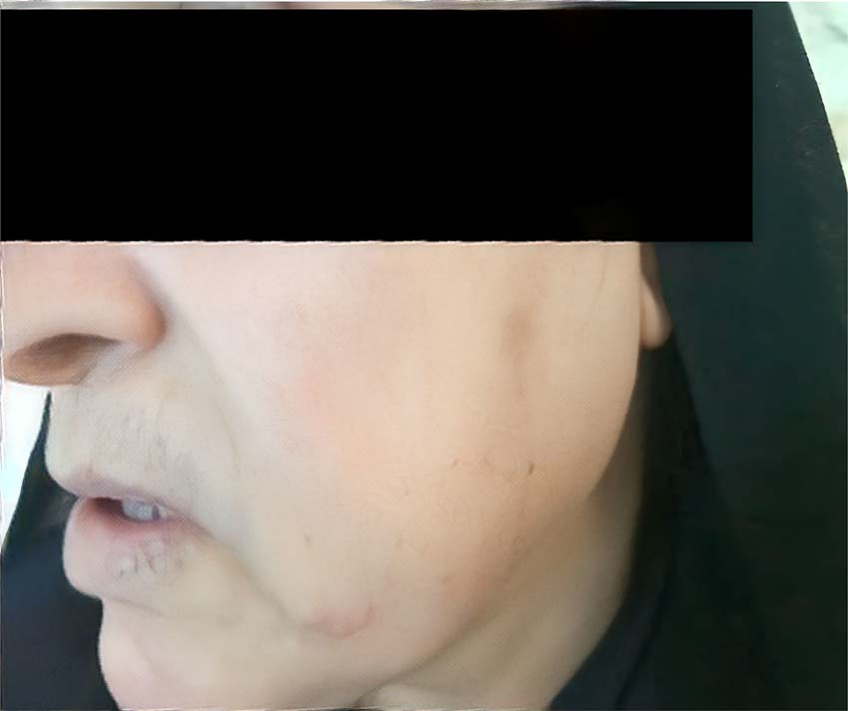
Photograph of the patient showing left parotid gland enlargement.

Physical examination showed bilateral parotid gland enlargement (Figs. 1 and 2).

Her treatment included amlodipine 10 mg daily, atorvastatin 10 mg daily, carvedilol 12.5 mg twice daily, torsemide 40 mg daily, levothyroxine 100 mcg daily, and omeprazole 20 mg daily.

Laboratory analysis revealed an increase in creatinine from 1.1 to 2.12 in a year, anemia with hemoglobin 8.2 g/dl, and a blood urea nitrogen (BUN) level of 108 mg/dl. Free T4, Free T 3,and thyroid stimulating hormone(TSH) tests were normal. Thyroid peroxidase antibody (Anti TPO) was negative. Urinalysis was normal. Serum and urine protein electrophoresis were negative for monoclonal proteins. Immune-profile tests including anti-nuclear antibodies, Anti-La, Anti-Smith, anti-ds DNA antibodies, Anti-Cytoplasmic Citrulinated peptide antibody, Rheumatoid factor, P-ANCA,C-ANCA, anti-Ro were negatives. Virology tests were negative. Thyroid ultrasound showed decreased vascularity of patchy, ill-defined hypoechoic areas in both lobes with involvement of the thyroid parenchyma.

**Figure 3:**
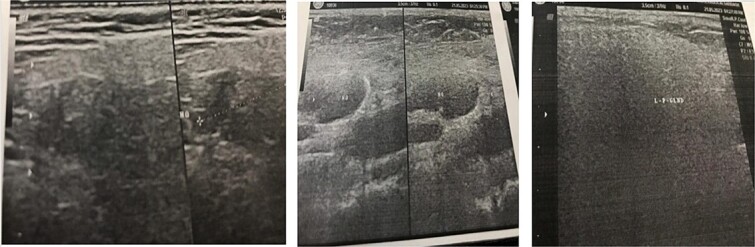
Sonographic findings of IgG4-related disease of both parotid gland.

**Figure 4:**
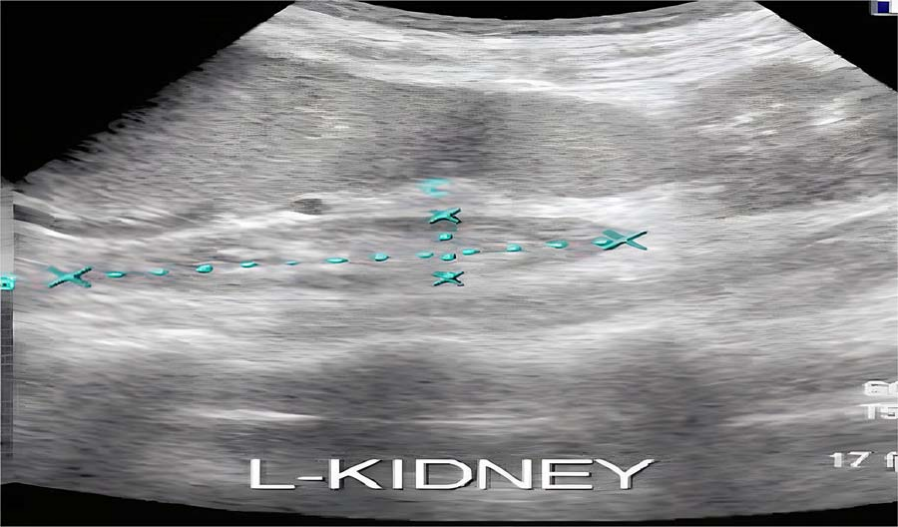
Ultrasound of the kidney showing an atrophic left kidney.

Ultrasound of the parotid gland has been made. (Fig. 3). Ultrasound of the kidney showed an atrophic left kidney (Fig. 4).

**Figure 5:**
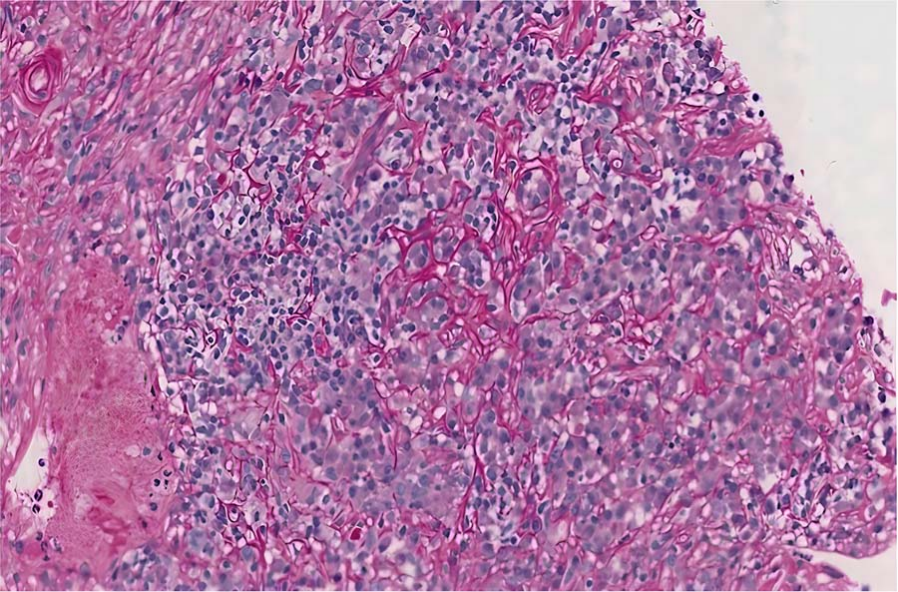
Left parotid gland biopsy revealing lymphoplasmacytic infiltration and fibrosis with IgG4+ plasma cells.

Left parotid gland biopsy was performed revealing lymphoplasmacytic infiltration and fibrosis with IgG4+ plasma cells (Fig. 5). Two serial sections were used for immunohistochemical evaluation. One slide was stained for IgG4+ plasma cells (antibody from The Binding Site, San Diego, Ca, 1∶15 000 dilution) and the other for IgG+ plasma cells (antibody from Dako, 1∶10 000 dilution). Immunostaining used Ventana automated instruments with Ultraview detection (Ventana, Tucson, AZ). The pathologist counted the numbers of IgG4+ cells in three high power fields (hpf, 400X, about 0.3 mm2) and the IgG+ cells in the corresponding fields of the paired slides. These data are expressed as the mean number of positive cells per hpf.

A percutaneous renal needle biopsy showed interstitial fibrosis and tubular atrophy, inflammatory cell infiltration and an increased number of plasma cells. There were clusters with more than 12 IgG4-positive plasma cells per high-power field detected by immunoperoxidase staining. Serum IgG4 levels were elevated at 402 mg/dl.

Based on the above findings, IgG4-RKD was diagnosed [2, 4, 5].

She was treated with 1 mg/kg/day of oral prednisone, and azathioprine. Unfortunately, 2 weeks later, her creatinine worsened to 2.98 mg/dl.

Three months later, she presented to the emergency department due to dyspnea, and fatigue, and she was taken40mg/day prednisone, and 75 mg/day Azathioprine. Physical examinations showed pallor.

Laboratory tests were as follows: Hg 7.3 g/dl, BUN 81 mg/dl, Cr 7.3 mg/dl, CO2 14, anion gap 16, Cl 111 mg/dl, Ca 8.3 mg/dl, ESR of 14 mm/hr. Urine protein of 34 mg, urine creatinine of 25.72 mg/dl, and urine protein/creatinine ratio of 0.8 mg/mg. IgG4 level of 139 mg/dl.

Renal ultrasound revealed an atrophic left kidney, and a new lobulated appearance of the right kidney compared to imaging from six months prior. She was treated with 500 mg intravenous methylprednisolone, sodium bicarbonate infusions, electrolyte replenishment, and blood transfusion. Creatinine level decreased to 5.9 mg/dl. At the time of discharge, she was taking sodium bicarbonate (1200 mg) three times daily, prednisone (60 mg) daily, calcium acetate (500 mg) three times daily, and potassium chloride (20 mEq) daily.

Two weeks later, she was readmitted with symptoms of weakness and edema of the lower extremities. Her laboratory markers again demonstrated a BUN of 112 mg/dl and a Cr of 6.8 mg/dl. Hemodialysis was initiated, due to the uremic symptoms, and progressing renal insufficiency. No signs of renal recovery were observed for 3 months, and the patient had died during hemodialysis, due to cardiac arrest. No post mortem was done.

## Discussion

The exact prevalence of IgG4-related disease is not known, and it affects middle-aged and older males [2]. In this case, the patient is a 68-year-old female.

IG4-related disease is a multi-organ, fibro-inflammatory status with tumefactive lesions of unknown etiology and characteristic histopathological features. Virtually any organ may be involved, but the most frequently involved organs are the pancreas, kidneys, salivary glands, orbital structures, and retroperitoneum [1, 2]. Tubulointerstitial nephritis with an abundance of IgG4+ plasma cells and storiform fibrosis located in the interstitium is the common feature of renal involvement [6]. Renal involvement is widely different, and often accompanied by extra-renal lesions such [2, 7]. In our case, our patient had evidence of extra-renal manifestations of IgG4-RD. A key finding in cases of IgG4-RKD is the presence of storiform fibrosis, which was in our case.

Many patients with IgG4-RD may have no signs or symptoms for months or even years before the diagnosis is made, and the disease can often appear as a mass, but other symptoms including fatigue, weight loss, jaundice, abdominal pain, headaches eye bulging, dyspnea, and blockage of urine flow [1, 2, 6]. Our patient had no symptoms.

Laboratory findings included: Elevated serum IgG 4 levels of more than 1.4 g/l are seen in 70% to 80% of patients. In our case, IgG4 titers were four times above the normal limit.. Normal levels do not exclude this disease [2, 6]. Elevated total IgG and IgE, peripheral eosinophilia, ESR (Erythrocyte Sedimentation Rate), and C -reactive protein were found. Aanti-nuclear antibody titers are positive in 50% of cases, elevated rheumatoid factor in 20% of patients, and hypocomplementemia were also reported [2]. Our patient had normal complement levels. PCR of the IgG4:IgG RNA ratio, serum IgG4 to total IgG ratio, and multicolour flow cytometry have been suggested as new diagnostic laboratory markers [8–10]. In our case, no history or clinical features and laboratory markers, including renal biopsy, indicate the other causes that were mischaracterized with this disease.

Radiological images cannot differentiate between malignancy and benign disease in the affected organs [6].

Diagnosis is based on clinical and histological features. There are criteria available for the diagnosis, such as the 2019 ACR/EULAR classification criteria for IgG4-RD [4, 11], as our patient was diagnosed.

Malignancy, infection, and some autoimmune diseases may be mischaracterized with this disease [2, 9, 10].

Glucocorticoids are the first line of therapy, leading to improvement in creatinine levels [5]. Because of the frequent disease relapse after glucocorticoids are stopped, and glucocorticoids side effects have prompted a search for other effective options such as methotrexate/azathioprine/mycophenolate mofetil to prevent further scarring and organ damage [1, 5].

Rituximab is approved in refractory cases to steroids. RTX is effective for both induction therapy and treatment of relapses in IgG4-RD, but relapses are frequent after B-cell reconstitution. Maintenance therapy with systematic RTX infusions is associated with longer relapse-free survival and might represent a novel treatment strategy. Baseline high disease activity and the lack of maintenance therapy with RTX are risk factors of relapse. Infection and hypogammaglobulinemia were reported after RTX treatment in a few patients [12].

In all cases, there was an improvement in renal function as measured by a decrease in serum creatinine or eGFR [2]. In regards to our patient, she was initially treated with steroids, azathioprine, and dialysis due to worsening renal function. Takako Saeki, et al retrospectively reported 43 patients with IgG4-related kidney disease, and 34 were treated with, and maintained on corticosteroids. One month after the start of treatment, most of the abnormal parameters had improved. In 34 of the steroid-treated patients whose follow-up period was more than 12 months, the estimated glomerular filtration rate (eGFR) before treatment did not differ [6].

## The novelty of our case

Rare cases observing the progression to end-stage kidney disease from IgG4-RD were found, and our case is the first one in which the patient had died in a short period (3 months later after follow-up).

The limitation is that it’s a case report, the findings, and the aspects of the patient presentation and progression may not apply to other cases, may not be generalizable to all patients with Ig G4-RD.

## Conclusion

However, the rarity and severity of our case might not present the typical course of Ig G 4-RD, early detection of IgG4-RKD provides the best chance for preserving renal function [10, 11], Unfortunately, our patient had died. We wanted to share this case with clinicians managing similar cases.
